# Evaluating women’s acceptability of treatment of incomplete second trimester abortion using misoprostol provided by midwives compared with physicians: a mixed methods study

**DOI:** 10.1186/s12905-022-02027-y

**Published:** 2022-11-05

**Authors:** Susan Atuhairwe, Claudia Hanson, Lynn Atuyambe, Josaphat Byamugisha, Nazarius Mbona Tumwesigye, Ronald Ssenyonga, Kristina Gemzell-Danielsson

**Affiliations:** 1 Department of Obstetrics and Gynaecology, Makerere University, Kampala, Uganda; 2 Department of Reproductive Medicine and Infertility, Mulago Specialised Women and Neonatal Hospital, Kampala, Uganda; 3 Department of Public Health Sciences, Karolinska Institutet, Solna, Sweden; 4 Department of disease control, London School of Hygiene and Tropical Medicine, London, UK; 5 Department of Community Health and Behavioural Sciences, School of Public Health, Makerere University, Kampala, Uganda; 6 Department of Epidemiology & Biostatistics, School of Public Health, Makerere University, Kampala, Uganda; 7 Department of Women and Children’s Health, Karolinska Institutet, Stockholm, Sweden; 8 WHO Collaborating Centre, Karolinska University Hospital, 17176 Stockholm, Sweden

**Keywords:** Acceptability, Task sharing, Incomplete abortion, Post abortion care, Misoprostol, Second trimester, Uganda

## Abstract

**Background:**

Studies evaluating task sharing in postabortion care have mainly focused on women in first trimester and many lack a qualitative component. We aimed to evaluate patient acceptability of treatment of incomplete second trimester abortion using misoprostol provided by midwives compared with physicians and also gained a deeper understanding of the patients’ lived treatment experiences in Uganda.

**Methods:**

Our mixed methods study combined 1140 structured interview data from a randomized controlled equivalence trial and in-depth interviews (*n* = 28) among women managed with misoprostol for second trimester incomplete abortion at 14 public health facilities in Uganda. Acceptability, our main outcome, was measured at the 14-day follow-up visit using a structured questionnaire as a composite variable of: 1) treatment experience (as expected/ better than expected/ worse than expected), and 2) satisfaction - if patient would recommend the treatment to a friend or choose the method again. We used generalized mixed effects models to obtain the risk difference in acceptable post abortion care between midwife and physician groups. We used inductive content analysis for qualitative data.

**Results:**

From 14th August 2018 to 16th November 2021, we assessed 7190 women for eligibility and randomized 1191 (593 to midwife and 598 to physician). We successfully followed up 1140 women and 1071 (94%) found the treatment acceptable. The adjusted risk difference was 1.2% (95% CI, − 1.2 to 3.6%) between the two groups, and within our predefined equivalence range of − 5 to + 5%. Treatment success and feeling calm and safe after treatment enhanced acceptability while experience of side effects and worrying bleeding patterns reduced satisfaction.

**Conclusions:**

Misoprostol treatment of uncomplicated second trimester incomplete abortion was equally and highly acceptable to women when care was provided by midwives compared with physicians. In settings that lack adequate staffing levels of physicians or where midwives are available to provide misoprostol, task sharing second trimester medical PAC with midwives increases patient’s access to postabortion care services.

**Trial registration:**

ClinicalTrials.gov NCT03622073.

**Supplementary Information:**

The online version contains supplementary material available at 10.1186/s12905-022-02027-y.

## Plain English summary

Approximately 9.6% of abortion-related deaths occur in Sub-Saharan Africa. These deaths can be prevented if unintended pregnancies are avoided, women can access safe abortions within the expectations of the country’s laws, and post abortion care (PAC) services are provided equitably. Previous research shows that women with abortion complications in the first trimester of pregnancy can be treated with misoprostol by either midwives or physicians. This sharing of tasks between the midwives and physicians is safe, effective, and acceptable. However, there is a gap in evidence on task sharing in the second trimester. To check practicability of task sharing in second trimester, we aimed to evaluate patient acceptability of treatment of incomplete second trimester abortion using misoprostol provided by midwives compared with physicians and also gained a deeper understanding of the patients’ lived treatment experiences. Our study therefore combined quantitative and qualitative approaches. Women’s acceptability of misoprostol treatment for incomplete second trimester abortion was found to be equally acceptable when provided by midwives compared with physicians. Treatment success, feeling calm and safe after treatment increased acceptability, while experience of side effects and worrying bleeding patterns reduced satisfaction. Counselling of women may address some of these problems since it provides reassurance and reduces anxiety. In settings that lack adequate staffing levels of physicians or where midwives are available to provide misoprostol, task sharing second trimester medical PAC with midwives increases patient’s access to PAC services.

**Supplementary Information:**

The online version contains supplementary material available at 10.1186/s12905-022-02027-y.

## Background

Unsafe abortion is the fourth leading cause of maternal mortality globally, and 9.6% of abortion-related deaths occur in Sub-Saharan Africa [1]. Deaths from unsafe abortion are largely preventable if unintended pregnancies are avoided, women can access safe abortions within the confines of the country’s laws, post abortion care (PAC) services are provided equitably, and contraceptive services widely available [2]. PAC is described as the comprehensive treatment of women following spontaneous or induced, safe or unsafe abortion [3]. To maximize gains from emergency treatment for women seeking care at health facilities, PAC services should be safe, effective, and acceptable. A recent literature review by Ngalame and colleagues showed that task sharing among physicians and mid-level health cadres for first trimester medical PAC is safe, effective, and also acceptable to both women and health care providers [4]. However, a key gap revealed was the lack of evidence on task sharing in the second trimester. Of note, women in second trimester are more at risk of dying from abortion complications compared to those in first trimester [5]. The gap is even worse for young women and rural women [4]. It is anticipated that lessons learnt from first trimester PAC task sharing can be extrapolated to second trimester PAC as well. Midwives once trained can competently manage uncomplicated PAC patients with misoprostol [2, 6]. This reduces delays at health facilities to initiate appropriate care hence less progression to more severe complications, and secondly relieves physicians to perform other tasks especially in contexts where they are limited in number [4].

Previous evidence on PAC considered acceptability as a sub-study of a large randomized controlled trial (RCT) with very few studies employing qualitative methods and hardly any done in the second trimester [7, 8]. Traditionally, the measure of acceptability among women has been collected through client exit interviews at the two-weeks follow up visit using standardized questionnaires [7, 8]. This means that the client’s unbiased perceptions of the intervention (intrinsic characteristics and available options) are not captured which may later on be influenced by the treatment experience. On the other hand, satisfaction with the intervention is closely related to the success of the treatment outcome, so it’s likely to be higher if the client achieves a successful treatment outcome [9]. Studies have also shown that acceptability is likely to be high if clients are given an opportunity to choose the treatment allocation arm although this is not the conventional methodology used in RCTs [10].

Several PAC studies use different parameters to measure acceptability: comparison of treatment with previous abortion experience, treatment experience in relation to expectations, recommendation of the same method to a friend, and future preference of same method where the last two are taken as measures of satisfaction [11, 12]. A lack of qualitative studies in previous work on acceptability of misoprostol for PAC leaves a gap in understanding deeply the characteristics that may influence values, perceptions and satisfaction [4, 7]. Although not often used in RCTs, mixed methods provide a more complete picture than a standalone quantitative or qualitative study since it integrates benefits of both research paradigms [13]. Mixed methods also offer enormous potential for generating new ways of understanding the complexities and contexts of social experience, and for enhancing our capacities for social explanation [14]. Researchers also utilize mixed methods to provide answers as to why an intervention worked or didn’t work, triangulation, and implementation fidelity [15].

In a country like Uganda where an estimated 130,000 women experience abortion-related complications annually [16], and abortion-related maternal deaths are 8 % [17], task sharing is an important strategy for equitable service delivery. PAC services in Uganda are available from primary level health facilities up to referral hospitals. First trimester PAC is offered at all pubic health care facilities; however, second trimester PAC is only accessed at Health centre IVs and hospitals where physicians are available [18]. The current Ministry of Health policy in Uganda allows for midwives to manage first trimester PAC patients either with MVA or misoprostol; however, sharp curettage is used for uterine evacuation in the second trimester and is restricted to physicians [18]. Although second trimester PAC with misoprostol by physicians is currently not part of the Uganda clinical guidelines, literature shows that second trimester PAC with misoprostol can be highly effective [6, 19, 20]. Involvement of midwives in PAC is a recognized high impact practice for improved quality services especially in low-income settings where physicians are scarce [21]. To check feasibility of task sharing in second trimester medical PAC, we aimed to evaluate patient acceptability of treatment of incomplete second trimester abortion using misoprostol provided by midwives compared with physicians and also gained a deeper understanding of the patients’ lived treatment experiences and have a better contextualization of the RCT results.

## Methods

### Study design

Our mixed methods study used secondary outcome data from a randomized controlled equivalence trial (RCT) and data from in-depth interviews (IDIs) with a sub-set of women who participated in the RCT [6]. The primary objective of the trial was to investigate effectiveness and safety of second trimester incomplete abortion treatment with misoprostol when managed by midwives compared to doctors [22]. The trial was performed and reported in accordance with CONSORT guidelines and registered at ClinicalTrials.gov on 9th August 2018, registration number NCT03622073. We used phenomenology as our methodological orientation to gain deeper insights in the patients’ lived experiences of their treatment in the RCT [14]. The checklist for consolidated criteria for reporting qualitative research guided the qualitative data presentation and reporting (COREQ) [23].

### Participants and setting

#### Quantitative

The trial was implemented in 14 public health facilities providing comprehensive emergency obstetric care services. The study sites included: two referral hospitals, eight general hospitals, and four health centre IVs in Uganda’s central region. We stopped recruitment of participants early at four study sites, three of which had slow enrollment and the fourth was turned into a covid treatment center. In regards to this acceptability study, we included all women participating in the RCT who were randomized, received treatment and returned for the 2 weeks’ follow-up visit [6]. We collected trial data from 14th August 2018 to 16th November 2021.

#### Qualitative

On accrual of over 50% of the trial sample size, we performed an interim analysis and obtained quantitative acceptability data that we used to identify participant attributes and respondents for the qualitative research.

We purposively selected a maximum variation sample of women based on: age (up to 24 years/ above 24 years), parity (up to four/ above four pregnancies), prior occurrence of abortion, those with medical treatment failure (negative cases), level of health facility (referral hospital/ general hospital/ health centre), and rural or urban location of health facility; so as to get a diversity of experiences. Using the generated sampling frame, the research team made a list of respondents with their telephone contacts and study sites, and scheduled appointments. Six women declined to participate due to: long distance from the health facility, work-related engagements, and lack of transport money despite being informed that they would receive a modest transport reimbursement. Participant recruitment occurred until the point of meaning saturation, at 28 respondents. Qualitative research data collection occurred at six study sites between May and June 2021.

### Data collection tools and measurements

#### Quantitative

One hundred and eighty-three trained health care providers (HCPs) comprised of doctors and midwives participated in the trial. Using a predefined checklist developed by the research team [22], trained midwives screened all women with abortion complications for eligibility. Research assistants obtained informed written consent from eligible participants and allocated them to the randomization groups, either midwife (intervention group) or doctor (control group) for clinical assessment and management. We previously reported other trial details [22]. Participants got pre-discharge information on danger signs and returned for a follow-up visit after 2 weeks.

Research assistants used pretested interviewer administered quantitative acceptability and satisfaction questionnaires to collect data. They gathered information on socio-demographic characteristics, reproductive history, and treatment outcome on the initial visit; then acceptability, side effects, pain, bleeding, unscheduled visits, and information received concerning treatment on the follow-up visit. All participants received a modest transport reimbursement at the follow-up visit.

#### Outcomes and measurements

We measured acceptability as a composite variable of: 1) treatment experience (as expected/ better than expected/ worse than expected), and 2) satisfaction - if patient would recommend the treatment to a friend (yes/no) or choose the method again (yes/no). Few studies consistently use the same parameters [7, 24, 25]; however, our parameters may offer a comprehensive measure of acceptability. We considered women satisfied if they would recommend the treatment to a friend or choose the method again. Women that did not recommend the treatment to a friend and not choose the method again were taken as not satisfied. We recorded patients as receiving acceptable PAC if the treatment expectations were as expected/ easier than expected and the patient was satisfied. We considered PAC as unacceptable if the treatment expectations were worse than expected or the patient was not satisfied.

Additionally, socio-demographic characteristics, reproductive history, side effects (composite variable of the presence of pre-specified symptoms labelled as - yes/no), pain, bleeding, unscheduled visits (yes/no), information received concerning treatment (yes/no), feeling calm and safe after treatment (yes/no), and treatment outcome were considered independent variables for acceptability. All variables except treatment outcome were self-reported. We measured the intensity of pain using a visual analogue scale with a zero-to-ten-point scale, zero showed no pain while ten showed the worst pain experienced. Bleeding during treatment was ascertained as the severity in comparison to normal menstrual bleeding (less than/ same as/ heavier than). Treatment outcome was either complete (patient was treated with misoprostol alone) or incomplete (patient required an additional surgical evacuation), and information was abstracted from the clinical record.

#### Qualitative

We interviewed respondents at the health facility ensuring privacy and confidentiality. We used a pilot tested interview guide with open-ended questions and probes to get a deeper understanding of the women’s lived PAC experience, perceived advantages and disadvantages of the treatment, how the treatment experience could have been made better, whether they would recommend or use the method again. Both English and Luganda languages were used based on the respondents’ preference. Two research assistants and the main researcher carried out the interviews, and held daily debrief meetings to discuss emerging ideas and also determine attainment of meaning saturation. The research process allowed for flexibility in the interview guide according to new views. We did not repeat any interviews. Each interview lasted 30 to 40 min and participants received a transport reimbursement. All interviews were tape recorded and the scribe took notes as well. We transcribed all the data and the main researcher read through all transcripts to ensure wholeness of the data. No transcript was returned to the respondents for correction or comment.

### Data management and data analysis

We performed sequential QUAN - QUAL analysis [15], that began with quantitative analysis followed by qualitative analysis. Our approach used the qualitative as explanatory for the quantitative.

#### Quantitative

We entered quantitative data into EpiData 3.1 and analyzed using Stata version 14. The data entry screen had been fitted with range and consistency checks. Final data cleaning was carried out using Stata.

We computed the risk difference in overall acceptability between the midwife and physician groups as the difference in proportion of participants in each group that found the treatment acceptable. We estimated the risk difference using a generalized linear mixed-effects model with the health-care facility as the random effect and treatment group as the fixed effect and confirmed equivalence if the risk difference and the 95% confidence interval (CI) of the estimate was within the − 5 to + 5% pre-defined range. *P*-values lower than 0.05 were considered statistically significant. To obtain the adjusted risk difference, we used the backward variable elimination technique beginning with co-variates: study arm, history of abortion, age, marital status, religion, education level, occupation, number of pregnancies, and number of live births. The adjusted risk difference was estimated as the predicted risk difference at the average of the covariates study arm, facility, religion, and education in the final model. We used the Bayesian information criterion from the likelihood-ratio test to assess for best model fit.

We also used the generalized linear mixed-effects model with random effect specified for the health-care facility and co-variates as fixed effects to obtain percentage differences in overall acceptability across demographic, reproductive and treatment experience factors. All factors with a *p*-value of < 0.2 at bivariate analysis were entered in the multivariable model. Elimination criteria was p-value > 0.05, except for unscheduled visits variable that stabilized the model. The final best fit model of factors independently associated with overall acceptability included: religion, complete abortion, bleeding during treatment, unscheduled visits, and feeling safe and calm after treatment variables. We compared participating PAC providers’ background experience before study start and did a sensitivity analysis on the women lost to follow-up in each study group.

#### Qualitative

We used inductive content analysis to get a deeper understanding of the women’s PAC acceptability. To obtain a sense of whole, transcribed text was read through several times, and a whole individual interview taken as the unit of analysis to preserve the context. The text was divided into meaning units, condensed and labelled with a code to constitute the manifest content, using HyperRESEARCH software version 4.5.0. Utilizing the inductive approach, we abstracted codes and grouped them into categories agreed upon by two researchers. This process was iterative. We formed a coding sheet reflecting code frequencies for the midwife and physician groups. An interpretation of the underlying meaning (latent content) was generated through discussion by formulating themes from the categories.

### Ethical considerations

We obtained ethical clearance from Makerere University School of Medicine Research and Ethics Committee (Rec ref. 2017–016) and the Uganda National Council for Science and Technology (HS153ES), and administrative clearance obtained from the participating health facilities. All study participants signed informed consent.

We submitted annual study progress reports to the ethical committees for ethical renewals.

## Results

### Socio-demographic characteristics of participants

#### Quantitative

**Fig. 1:**
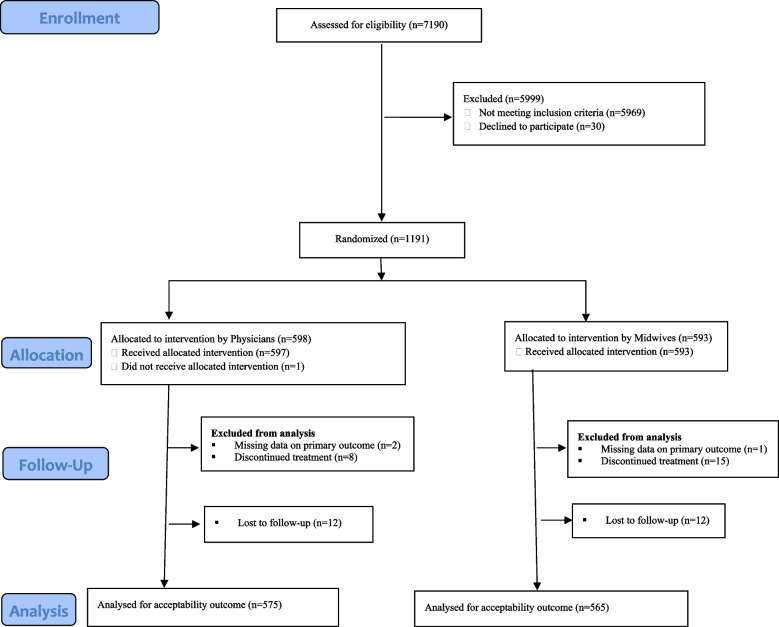
Trial profile

Of the 7190 women screened for eligibility between August 2018 and November 2021, we randomized 1191 women. We allocated 598 women to the Physician’s group and 593 women to the midwife’s group. Five hundred and eighty-seven women in the physician’s group and 577 women in the midwife’s group received the appropriate misoprostol treatment. We effectively followed up and captured acceptability data for 1140 women, 575 in the physician’s group and 565 in the midwife’s group. In each group, 12 women were lost to follow-up (Fig. 1).

With the exception of women lost to follow-up more likely to be of born-again or SDA religion, there was no statistically significant difference in the other sociodemographic and reproductive factors between women lost to follow-up and those that returned (results not shown).

The participants’ mean age in years was 26.2 (SD 6·1; range 15 to 47). A relatively higher proportion of women were married/cohabiting (77% [878/1140]), catholic (42% [478/1140]), and unemployed (47% [541/1140]). The median number of pregnancies was 3 (IQR 2 to 4), median number of live births 1 (IQR 0 to 3), and median gestational age of 14 weeks (IQR 13 to18). Only 19% (216/1140) of the women had a previous history of abortion. The participant’s socio-demographic and reproductive characteristics did not significantly differ between study groups (Table 1).

**Table 1:** Sociodemographic and reproductive history characteristics of participants by provider type

Characteristic	Midwife ***N*** = 565	Physician ***N*** = 575	Total ***N*** = 1140	***p***-value
**Mean age in years (SD)**	26.1 (6.8)	26.3 (6.0)	26.2 (6.1)	0.676*
**Marital status**				
Married or cohabiting	423 (75)	455 (79)	878 (77)	0.087^#^
Single/divorced/widow	142 (25)	120 (21)	262 (23)	
**Religion**				
Catholic	238 (42)	240 (42)	478 (42)	
Anglican	168 (30)	188 (33)	356 (31)	0.221^#^
Islam	121 (21)	99 (17)	220 (19)	
Other (SDA^a^, Born again)	38 (7)	48 (8)	86 (8)	
**Education**				
No formal	16 (3)	19 (3)	35 (3)	
Primary	256 (45)	239 (42)	495 (43)	0.636^#^
Secondary	236 (42)	256 (44)	492 (43)	
Tertiary	57 (10)	61 (11)	118 (10)	
**Occupation**				
Unemployed	267 (47)	274 (48)	541 (47)	
Formal employment	59 (10)	75 (13)	134 (12)	0.320^#^
Self employed	239 (42)	226 (39)	465 (41)	
**Median gestation age in weeks (IQR)**	14 (13–18)	14 (13–18)	14 (13–18)	0.492^^^
**Median number of pregnancies (IQR)**	3 (2–4)	3 (2–4)	3 (2–4)	0.436^
**Median number of live births (IQR)**	1 (0–3)	2 (0–3)	1 (0–3)	0.311^
**Previous history of abortion**				
Yes	109 (19)	107 (19)	216 (19)	
No	456 (81)	468 (81)	924 (81)	0.768^#^

Data are n (%) unless otherwise stated. ^a^*SDA* Seventh-day Adventist

Test statistic used - *t-test, #chi-square, ^Wilcoxon rank-sum test

#### Qualitative

Twenty-eight women participated in the IDIs between May and June 2021 (Additional file 1). The respondents purposively varied in age, parity, previous experience of abortion, treatment failure, health facility type and location. IDI respondents were slightly older than women in the quantitative group (mean age of 28 vs 26 years). Like in the quantitative group, most women were married. However, majority of the women were self-employed or in formal employment, practiced Islam, and had attained either primary or secondary education level.

### Misoprostol treatment is equally acceptable between midwives and doctors

Amongst 1140 women, 539 (95.4%) women in the midwife group and 537 (93.4%) women in the physician group reported the treatment experience either better than or same as expected (Table 2).

**Table 2:** Women’s acceptability of misoprostol treatment for incomplete abortion by provider

	Midwife n(%) ***N*** = 565	Physician n(%) ***N*** = 575	All women n(%) ***N*** = 1140
**Treatment experience compared with expectations**			
Better than expected	381 (67.4)	365 (63.5)	746 (65.4)
As expected	158 (28.0)	172 (29.9)	330 (29.0)
Worse than expected	26 (4.6)	38 (6.6)	64 (5.6)
**Patient would recommend the treatment to a friend**			
Yes	556 (98.4)	568 (98.8)	1124 (98.6)
No	9 (1.6)	7 (1.2)	16 (1.4)
**Patient would choose the method again**			
Yes	552 (97.9)	566 (98.4)	1118 (98.2)
No	12 (2.1)	9 (1.6)	21 (1.8)
**If patient was satisfied**^**a**^			
Yes	557 (98.6)	571 (99.3)	1128 (99.0)
No	8 (1.4)	4 (0.7)	12 (1.0)
**Overall Acceptability**^**b**^			
Acceptable	534 (94.5)	537 (93.4)	1071 (94.0)
Unacceptable	31 (5.5)	38 (6.6)	69 (6.0)

^a^Satisfied = Yes, if patient would recommend the treatment to a friend or choose the method again

^b^Overall Acceptability = Acceptable if the treatment expectations were “as expected/ easier than expected” and the patient was satisfied

In both groups, there was a similar distribution of women who would recommend the treatment to a friend (98.6%) or choose the method again (98.2%), and were therefore satisfied with the treatment (99%). Regarding overall acceptability, treatment was acceptable in 1071 (94%) women and unacceptable in 69 (6%) women. Five hundred and thirty-four (94.5%) women in the midwife’s group and 537 (93.4%) women in the physician’s group found the treatment acceptable, with a risk difference of 1.1% (95% CI, − 1.3 to 3.5%) between the two groups. The adjusted risk difference in the proportion of women whose treatment was acceptable between the midwife group and physician group was 1.2% (95% CI, − 1.2 to 3.6%) (Table 3), and did not cross the equivalence margins of − 5 to 5%. Hence, the overall acceptability did not significantly differ between the midwife and physician groups.

**Table 3:** Outcome of women’s overall acceptability by provider

Acceptability	Midwife	Physician	Risk difference (95% CI)	
			Unadjusted	Adjusted^**a**^
Randomized and received intervention	593	597	–	–
Successfully followed up	565	575	–	–
Overall acceptability	534 (94.5%)	537 (93.4%)	1.1% (−1.3 to 3.5%)	1.2% (−1.2 to 3.6%)

Data are n (%) unless otherwise stated

^a^Adjusted for facility, education (no formal vs primary, secondary or tertiary), and religion (Catholic vs Anglican, Islam or SDA/Born Again)

While our quantitative results showed equal acceptability between the midwife and physician groups, in the qualitative results, we found that most respondents who perceived HCPs as pleasant and responsible were treated by physicians. Both physicians and midwives were commonly described as caring and approachable. The former attribute was expressed by women of all age groups and the latter more in women above 24 years. Physicians were also found welcoming, merciful, and friendly. Some respondents treated by physicians at referral hospitals described the staff as hardworking and engaged since they monitored them closely even during off peak hours like at night and weekends. A few respondents however cited instances where HCPs were rude to patients and caretakers, particularly when they were tired and overwhelmed with work.

*The doctors didn’t treat me badly. They welcomed me, and were easy for me to approach. And whenever I told them about the pain I was feeling, they would care so much. (participant 20_28 years_referral hospital)*

*The truth is, the doctors … are not easy at all. Mostly when you go there with no connection. They are not easy. (participant 9_30 years_health centre)*

*But I think the doctor was also tired that day because there were so many people at ward that day and it was him who was being called all the time. (participant 1_20 years, general hospital)*

In a few instances, women parted with money to pay a HCP or buy some supplies like gloves that were lacking in the health facilities, and this was mentioned by women treated by physicians and midwives alike. This was more evident among those who eventually got a surgical evacuation.

*That one is a sure deal. They ask for it [money]. Yes, if you don’t have it, you will not receive the care as others who have. But this last time, he didn’t ask me for any money. (participant 9_30 years_health centre)*

*The only money we gave him was what he used to buy what he was going to use on me, but he didn’t ask us any money. We bought a polythene bag, gloves and other things which my mother bought while I was in the theatre. (participant 13_19 years_ health centre)*

### Treatment success is a key determinant of acceptable PAC

The prevalence of acceptable PAC was 21.8% (21.8, 95% CI 9.5 to 34.2%) greater among women who had a complete abortion using misoprostol alone than in women who required additional surgical evacuation (Table 4). In agreement, majority of participants interviewed who were managed efficiently and received complete healing were more likely to recommend treatment to their friends. Most respondents found the treatment adequate, timely, and strongly felt that it was life-saving. As a result, they were appreciative and happy with the treatment received. Following their successful treatment experience, most respondents would refer clients with the same problem since they had confidence that their friends would get good care at the public rather than private health facilities.

*There is a friend of mine who also miscarried. For her, she was lifting a jerrycan of water and I told her to also come here for treatment since the doctors here care for patients and treat them well. In fact, it is me who escorted her. I came with her; she was worked on and she got okay. And we went back. She was also given tablets [misoprostol]. (participant 14_26 years_health centre)*

*I had a friend who also had a miscarriage. But for her, she was washed [evacuated] several times. I told her that, I went to a government hospital, here, and they washed [evacuated] me once and I got okay. But for her, she had gone to a private hospital. She told me that she had been washed [evacuated] five times, even some days back. Even she has never gotten okay up to now. I told her to go to a government hospital than going in a private one. (participant 11_34 years_health centre)*

Most women at general hospitals and lower health facilities reported that their friends were only aware of surgical evacuation. Even after success with medical treatment, the friends would insist that they should return for surgical evacuation to ensure that the uterus was completely emptied. It was a surprise to them that one could completely heal with misoprostol alone.

*Yes, they [friends] were even happy about it. But they asked me why I was not washed in the stomach [evacuated]and they said I would have other problems since I was not washed [evacuated]. I told them that I wasn’t going to have any other problem. Since the type of work I do is heavy, I would have gotten those problems if they were meant to happen again. Yes, they even told me that I should come back and I get washed [evacuated]. " (participant 7_45 years_general hospital)*

In addition to one’s treatment experience and prior knowledge of misoprostol, a few respondents mentioned that the choice of a subsequent treatment method also depended on, the clinical condition, a short hospital stay, and a HCP’s decision after their thorough assessment.

*With those tablets [misoprostol], as long as you are not in a bad condition, you can be helped. But if you are in a very bad condition, the other method of being washed [surgical evacuation] helps you a lot. (participant 6_31 years_general hospital)*

**Table 4:** Women’s demographic, reproductive, and treatment experience by overall acceptability

	Acceptability		Total ***N*** = 1140	Bivariate analysis		Multivariable analysis	
	Acceptable ***N*** = 1071	Unacceptable ***N*** = 69		Unadjusted percentage differences^**c**^ (95% CI)	***P*** value	Adjusted percentage differences^**c**^ (95% CI)	***P*** value
**Religion**							
Anglican	338 (31.6)	18 (26.1)	356 (31.2)	Ref		Ref	
Catholic	456 (42.6)	22 (31.9)	478 (41.9)	0.5% (−1.9 to 2.8%)	0.71	−0.6% (−3.4 to 2.2%)	0.674
Islam	204 (19.0)	16 (23.2)	220 (19.3)	−2.2% (−7.8 to 3.3%)	0.435	− 2.0% (−7.5 to 3.5%)	0.478
Other (SDA^a^, Born again)	73 (6.8)	13 (18.8)	86 (7.5)	−10.1% (− 18.3% to − 1.9%)	0.016	−9.0% (− 15.4% to − 2.6%)	0.006
**Complete abortion**							
Yes	1017 (95.0)	47 (68.1)	1064 (93.3)	24.5% (9.8 to 39.2%)	0.001	21.8% (9.5 to 34.2%)	0.001
No	54 (5.0)	22 (31.9)	76 (6.7)	Ref		Ref	
**Experienced side effects**^b^							
Yes	229 (21.4)	38 (55.1)	267 (23.5)	−10.7% (−25.8 to 4.4%)	0.166	-^d^	-^d^
No	839 (78.6)	31 (44.9)	870 (76.5)	Ref		-^d^	-^d^
**Bleeding during treatment**							
Less than normal menses	618 (57.7)	22 (31.9)	640 (56.1)	Ref		Ref	
Same as normal menses	382 (35.7)	37 (53.6)	419 (36.8)	−5.4% (−8.7% to −2.0%)	0.002	−4.7% (−8.1% to −1.4%)	0.005
Heavier than normal menses	71 (6.6)	10 (14.5)	81 (7.1)	−8.9% (−18.7 to 0.9%)	0.074	−6.4% (− 15.8 to 3.0%)	0.180
**Unscheduled visits**							
Yes	22 (2.0)	7 (10.1)	29 (2.5)	−18.6% (−37.0% to −0.1%)	0.049	− 13.3% (−28.8 to 2.2%)	0.092
No	1049 (98.0)	62 (89.9)	1111 (97.5)	Ref		Ref	
**Received enough counselling**							
Yes	1066 (99.5)	67 (97.1)	1133 (99.4)	22.7% (6.0 to 39.3%)	0.008	-^d^	-^d^
No	5 (0.5)	2 (2.9)	7 (0.6)	Ref		-^d^	-^d^
**Felt calm and safe**							
Yes	1065 (99.4)	61 (88.4)	1126 (98.8)	51.7% (7.2 to 96.2%)	0.023	41.6% (3.3 to 80.0%)	0.033
No	6 (0.6)	8 (11.6)	14 (1.2)	Ref		Ref	

Data are n (column %) unless otherwise stated

^a^*SDA* Seventh-day Adventist

^b^Has three missing values

^c^These are percentage difference estimates for generalized linear models with gaussian family and identity link

^d^Not included in the final adjusted regression model

### Experience of side effects and worrying bleeding patterns reduce satisfaction

There was a trend in reduced PAC acceptability as the bleeding severity got heavier. Women whose bleeding pattern was the same as a normal menstrual period had 4.7% (− 4.7, 95% CI − 8.1% to − 1.4%) lesser prevalence of acceptable PAC than among women whose bleeding pattern was less than their normal menstrual period. PAC was also 41.6% (41.6, 95% CI 3.3 to 80%) more acceptable in women who felt calm and safe after treatment than among women who did not feel calm and safe after treatment. Irrespective of age and treatment group, most respondents in the IDIs perceived the treatment as safe even when some experienced a few side effects. Notably, 23.5% of participants in the quantitative experienced side effects, however side effects were not significantly associated with acceptability (Table 4). Pain was the commonly cited side effect and others included: misoprostol-related side effects, prolonged bleeding, prolonged duration in hospital, and perineal trauma for a few respondents who had received surgical evacuation. While majority of the respondents preferred misoprostol due to less pain, and fewer complications; a few preferred surgical evacuations since it was fast with a short hospital stay. Most women were afraid of surgical evacuation and the whole theatre experience, with many respondents mentioning that it carried higher risks of pain, infection, and injury to the genital tract. Participants’ medical and surgical management comparisons were based on either previous PAC experience or their perceptions from prior information.

*... But when I was given tablets[misoprostol], the pain was less but lasted long. Okay, with machines, it is painful but quick. And with tablets, it is less painful but slow to go away. When they are using machines on you, the pain is so much. At least when they gave me tablets, it was less painful and it is better to heal slowly than using machines which are so painful and heal quickly from the pain. (participant 21_23 years_referral hospital)*

Patients however received detailed explanations on the care given and what to expect, with provision for them to ask questions. This counselling provided great reassurance to patients and reduced their anxiety especially for those who initially thought they would have a surgical uterine evacuation.

*But the doctors first explained to me and told me that the tablets they were going to give me were going to do this and that and I heard that there were no things of using machines. (participant 20_28 years_referral hospital)*

Lastly, women whose religion was SDA/Born again had 9% (− 9, 95% CI − 15.4% to − 2.6%) less acceptable PAC than women who were Anglican. Whereas unscheduled visits and receiving enough information about the treatment were significantly associated with overall acceptability in the bivariate model, they were not statistically significant in the multivariable model.

## Discussion

### Main findings

This mixed methods study revealed that women’s acceptability of misoprostol treatment for incomplete second trimester abortion was equally acceptable when provided by midwives compared with physicians. We also demonstrate that treatment success, feeling calm and safe after treatment increase acceptability, while experience of side effects and worrying bleeding patterns reduce satisfaction. To our knowledge, this is the first mixed methods study addressing acceptability of medical PAC in the second trimester and the combination of quantitative and qualitative approaches provides opportunity to obtain a deeper and broader understanding of this phenomenon. Our study conducted across referral hospitals, general hospitals and health centre IVs reports a high acceptability regardless of the treatment group, making the results relevant in similar settings.

Our study reports equivalence in acceptable PAC among women treated by physicians or midwives which is similar to prior evidence from first trimester PAC [4, 7, 26]. Most HCPs in our study were already actively involved in first trimester PAC and received additional training [22]. This probably primed them with the requisite skills, competences, and attitudes to care for PAC patients which could explain our findings. In addition, our high overall acceptability of 94% is similar to previous first trimester PAC studies that report acceptability in the ranges of 92 to 97% [7, 8, 26]. Whereas our study used a composite outcome variable that combined - treatment experiences in relation to expectations, recommendation to a friend, or use of method again; prior studies utilized varying definitions of acceptability which might explain the slight variations in the estimates [7, 25, 26]. Our assumption is that the composite variable may provide a more holistic definition of acceptability since it combines aspects of women’s personal values and perceptions of the attributes of the treatment, quality of the treatment, and the health delivery system for the patient. Our qualitative interviews further report the HCPs’ positive attributes that might provide explanation for the acceptable PAC. Women perceived physicians as more pleasant and caring than midwives. We had minimal mention of rudeness for both cadres in our study although previous studies report an increased tendency of rudeness among the nurse-midwives [27, 28]. This might explain the more favored responses for the physicians rather than the midwives. Even when respondents reported rudeness, they rationalized it against the background of HCP’s heavy workload and therefore at some level found this understandable [28].

At times, women were required to pay money either directly to the HCP or for purchase of missing drugs and supplies. Our study agrees with previous evidence that demonstrates frequent stock-outs of essential drugs, supplies and commodities within Uganda’s health care system [4]. This may arise from poor health care financing by the government, inadequate forecasting at the health facility planning level, insufficient delivery of requested supplies, or artificial shortage at the health facility point due to theft or hoarding of drugs and supplies [27–29]. One respondent for example gave a scenario where the spouse had to pay for a drug that was already available at the health facility.

About 99% of the patients would either recommend the treatment to a friend or use the method again. Treatment success was a key determinant of acceptable PAC. Like in previous studies, women are more likely to recommend the same treatment to their friends if they have had a positive experience [7]. One outstanding phenomenon was that women perceived the treatment as life-saving. PAC is recognized as an essential component of both basic and comprehensive emergency obstetric care services [21]. Patients therefore appreciated that the health care system treated them as emergencies which increased their confidence in the public health facilities even denoting, they provided the true diagnosis and prescriptions rather than the private health facilities.

According to the Ministry of Health policy in Uganda, first trimester PAC is currently managed by medical or surgical evacuation while second trimester PAC is mainly treated with surgical evacuation [18]. However, like in previous studies, the public is still ignorant about medical PAC as most women approached the health facilities anticipating surgical evacuation [27]. Women got this information either from friends or prior experience. Nevertheless, the few women aware of misoprostol from previous first trimester PAC experience were more receptive and would choose the same treatment modality again. While history of previous abortion was not statistically significantly associated with acceptability, we did not obtain information on whether those abortions had been managed with medical or surgical modalities, and thus were unable to extrapolate the link between previous treatment options and overall acceptability quantitatively.

It is assumed that having a variety of treatment options increases one’s choice and autonomy [28]; however there were some women in the IDIs still inclined to the HCP making the decision for them. While the HCPs are more informed, we expect that if women are given the right counselling and explained the pros and cons of each treatment modality, they should have autonomy in decision making. Our IDI respondents were educated and had a stable income source, so it could reflect an inherent lack of autonomy in decision making or an acceptance of messaging from HCPs.

We noted a reduction in acceptability as vaginal bleeding during treatment got heavier. During medical PAC, we expect women to bleed as they expel the retained products [21]. Some women described this bleeding as prolonged compared to the surgical evacuation that takes a shorter time, which agrees with findings from prior misoprostol studies [9]. Almost 25% of women in the quantitative study experienced side effects. Although side effects were not significantly associated with acceptability in our study, they were frequently mentioned in the qualitative interviews. Even with the common side effects, women still felt calm and safe after treatment; probably due to the good counselling they had received. Unlike a Nigerian study [11], our quantitative results did not show statistical significance between information sharing and acceptability. However, the counselling women received may have reassured them and reduced their anxiety which could explain their feeling calm and safe after treatment despite experiencing side effects. Undesired effects of misoprostol were perceived to be less than those of surgical evacuation depicting medical treatment as more acceptable than surgical treatment [4], and is one main reason for WHO recommending medical options over surgical options for uncomplicated PAC patients [21]. We also found religion significantly associated with reduced overall acceptability. A possible explanation for this is that women’s reproductive choices are strongly influenced by interpretations of their religious doctrine [30].

Our study results have potential to contribute to revision of Ministry of Health PAC policy and treatment guidelines in Uganda. In a country where second trimester PAC has been in the docket of physicians, task sharing of medical PAC for uncomplicated cases with midwives may improve access to PAC services particularly for the rural population.

### Strengths and limitations

One strength of this study is the mixed methods approach that we used to generate convergent and divergent results, with the qualitative (IDIs) explanatory for the quantitative (RCT). Other strengths of the study are the large dataset, prospective data collection that ensured completeness of information, and our comprehensive definition of acceptability. A limitation of the study is that we only assessed acceptability for women who returned for the follow-up visit. However, the loss to follow-up was very minimal (2%) and except for religion, the other sociodemographic and reproductive factors did not significantly differ between women in the acceptability study and those lost to follow-up. While we do not expect any effect on the risk difference in proportions of overall acceptability between the physician and midwife groups, there may be an effect on the exact proportion of women with acceptable PAC. Secondly, all women in the quantitative and qualitative studies were interviewed at the health facilities. This could have prompted them to give more favored responses. We used trained and experienced research assistants and interviewers, standardized questionnaires, and flexible interview guides that allowed for probing to elicit a more comprehensive understanding of the women’s experiences. Thirdly, we tested acceptability among women randomized to HCPs who worked in close proximity at the PAC sites, which might bring about convergence in the outcome. To minimize this, we conducted regular supervisory visits to ensure adherence to the study protocol. Fourthly, we were unable to blind participants to the treatment allocation given the nature of the study. It is true that patients at times may not be able to distinguish the type of cadres. Of note, we selected participants for the qualitative interviews from women that had been treated by each group. The comparisons in the qualitative data matrix were also based on responses from the two groups.

### Methodological considerations

We used a maximum variation sampling technique based on the quantitative attributes for a rich collection of data. We considered each IDI as a unit of analysis to preserve the context, and conducted interviews until a point of meaning saturation when no new ideas emerged. A thick description of the data was provided in the results presentation which enhanced credibility. We carried out interviews in either English or Luganda languages based on the respondents’ preferences. Luganda is the commonly spoken local language in the central region. The interviewers and data transcribers were fluent in both languages to ensure correct translation and understanding of the information especially when Luganda was used. The main researcher (SA) is a Ugandan female obstetrician and gynaecologist, with over 10 years of research experience in PAC. She closely worked with two other male research assistants that have extensive experience in maternal health research. Respondents freely shared information with the research team. We used a pre-tested interview guide and allowed for flexibility based on emerging ideas and discussions with the research team during the debrief meetings. We read through transcripts many times to ensure reflexivity and discussed study findings with other qualitative researchers to enhance dependability. We carried out this research at rural and urban health facilities in the central region of the country, that offer comprehensive emergency obstetric care services, so transferability may be possible in settings with similar context.

## Conclusions

This mixed-methods study shows that misoprostol treatment of uncomplicated second trimester incomplete abortion was equally and highly acceptable when care was provided by midwives compared with physicians. We found that treatment success and feeling calm and safe after treatment enhanced acceptable PAC while experience of side effects and worrying bleeding patterns reduced satisfaction. Client counselling may address some of these shortfalls since it provides reassurance and reduces anxiety. Shortages of supplies, extortion of money especially for surgical services in Uganda are still challenges. In Uganda and similar settings that lack adequate staffing levels of physicians or where midwives are available to provide misoprostol, task sharing second trimester medical PAC with midwives increases patient’s access to PAC services. Also, in settings where midwife-provided care could be preferable, our study adds the value of expanding midwife-provided second trimester PAC (and other services) which can have an important influence on the level of health care setting where care is provided and access in a wider range of health care settings.

## Supplementary Information

**Additional file 1.** In depth-interview respondents’ characteristics.

## Data Availability

All data generated or analysed during this study are included in this published article [and its supplementary information files].

## Abbreviations

CI: Confidence interval
HCPs: Health care providers
IDIs: In-depth interviews
IQR: Interquartile range
RCT: Randomized controlled trial
PAC: Post abortion care
SD: Standard deviation
