# Supplementary material for: Evaluating women’s acceptability of treatment of incomplete second trimester abortion using misoprostol provided by midwives compared with physicians: a mixed methods study
Source: BMC Womens Health. 2022 Nov 5;22:434. doi: 10.1186/s12905-022-02027-y (PMC9637300; doi:10.1186/s12905-022-02027-y)
Supplement: Supplementary file 1 — Additional file 1. In depth-interview respondents’ characteristics. [file 12905_2022_2027_MOESM1_ESM.docx]

| **Unique ID** | **Age** | **Education level** | **Religion** | **Marital status** | **Occupation** | **Type of Health facility** |
| --- | --- | --- | --- | --- | --- | --- |
| 1 | 20 | Secondary | Anglican | Married | Housewife | General Hospital |
| 2 | 24 | Primary | Born again | Single | Hair dresser | General Hospital |
| 3 | 39 | Primary | Catholic | Married | None | General Hospital |
| 4 | 19 | Secondary | Moslem | Married | Student | General Hospital |
| 5 | 23 | Secondary | Catholic | Married | Retailer | General Hospital |
| 6 | 31 | Primary | Born again | Married | Farmer | General Hospital |
| 7 | 45 | None | Moslem | Married | Farmer | General Hospital |
| 8 | 29 | Secondary | Moslem | Married | None | General Hospital |
| 9 | 30 | Secondary | Anglican | Married | Businesswoman | Health centre IV |
| 10 | 25 | Primary | Catholic | Married | Businesswoman | Health centre IV |
| 11 | 34 | Primary | Born again | Separated | None | Health centre IV |
| 12 | 31 | Tertiary | Born again | Married | Pharmacist | Health centre IV |
| 13 | 19 | Primary | Anglican | Married | Farmer | Health centre IV |
| 14 | 26 | Primary | Moslem | Married | Farmer | Health centre IV |
| 15 | 22 | None | Moslem | Married | None | Health centre IV |
| 16 | 27 | Secondary | Catholic | Married | Farmer | Health centre IV |
| 17 | 35 | Tertiary | SDA^a^ | Married | Politician | Health centre IV |
| 18 | 38 | Primary | Catholic | Separated | Businesswoman | Referral hospital |
| 19 | 22 | Tertiary | Moslem | Married | Teacher | Referral hospital |
| 20 | 28 | Secondary | Moslem | Married | None | Referral hospital |
| 21 | 23 | Secondary | Born again | Married | Secretary | Referral hospital |
| 22 | 38 | Primary | Moslem | Married | Businesswoman | Referral hospital |
| 23 | 30 | Tertiary | Moslem | Married | Businesswoman | Referral hospital |
| 24 | 33 | Tertiary | Moslem | Married | Businesswoman | Referral hospital |
| 25 | 23 | Secondary | Moslem | Married | Housewife | Referral hospital |
| 26 | 34 | Secondary | Moslem | Married | Businesswoman | Referral hospital |
| 27 | 24 | Tertiary | Born again | Married | Businesswoman | Referral hospital |
| 28 | 28 | Primary | Catholic | Separated | Businesswoman | Referral hospital |

**Additional file 1 In depth-interview respondents' characteristics**

^a^SDA-Seventh-day Adventist
